# How to Choose a Mentor?

**DOI:** 10.5005/jp-journals-10008-1150

**Published:** 2013-09-06

**Authors:** Shibal Bhartiya, Parul Ichhpujani

**Affiliations:** Consultant, Department of Ophthalmology, Fortis Memorial Research Institute, Gurgaon, Haryana, India; Assistant Professor, Glaucoma Facility, Department of Ophthalmology, Government Medical College and Hospital, Chandigarh, India

**Keywords:** Mentor, Glaucoma, Career, Ophthalmology.

## Abstract

Mentorship programs have been found to be of great use in all career fields, especially medicine. Given that the practice of medicine is best learnt by a hands-on training, a mentorship program can prove invaluable for the young clinician scientist. A structured super-speciality training program can help in both, acquisition of clinical and research skills, as well as career growth.

**How to cite this article:** Bhartiya S, Ichhpujani P. How to Choose a Mentor? J Current Glau Prac 2013;7(3):128-129.

The word ‘Mentor' has its roots in Greek mythology. Mentor was the son of Heracles and Casopis. In his old age Odysseus placed mentor in charge of his son Telemachus, when he left for the Trojan War. When Athena, the goddess of heroic endeavor visited Telemachus she took the disguise of mentor to hide herself from the suitors of Telemachus' mother, Penelope. As mentor, the goddess encouraged Telemachus to stand up against the suitors and to find out what happened to his father. Because of mentor's relationship with Telemachus, and the disguised Athena's guidance for dealing with dilemmas, the name ‘mentor' has been adopted in English as a term referring to a person who imparts knowledge and shares his/her experience with someone less experienced.

Plutarch has rightly said,

‘The mind is not a vessel to be flled, but a fre to be kindled.'

A good mentor helps kindle this fre, helps one set a new career path, or find greater fulfllment in their chosen direction. In the present era, a mentor can also help network, and introduce his/her protege to opportunities and people that he/she would not otherwise have access to. A mentor provides guidance and inspiration, and can be a highly credible reference for grants, awards and professional positions.

Mentoring is increasingly viewed as a key factor contributing to a successful career in all fields including academic medicine,^1-15^ and has been found to be imperative for facilitating a young medical professional's career advancement and acquisition of clinical and research skills.^3-5,15^

When you decide to go in for a mentorship program it is important that you are clear about what it is that you want in your mentor, and what kind of help and advice you are seeking. These guidelines will at best help you narrow down your choice.

## PERSONALITY CREDIBILITY

It is essential to choose someone you can respect, and of course, that this person has the necessary expertize in your specific field. It also helps if you admire this particular person for his or achievements, because very often, you will have to follow your mentor's advice against your better judgment.^13^

Also, speak to past mentees, and how they found their experience with the particular person. In case you are not ready to become a clone, look for a mentor who will direct you toward your goals.

You do not need to find someone who has achieved the most in his or her field: just someone who has enough generosity of spirit to let you learn from their past experiences. You will, however, find that most successful people have this quality and are secure enough to give you credit whenever its due. Again, speaking to those who have been mentored will provide you enough insight to arrive at an informed decision.

## ACCESSIBILITY

It is essential that your mentor be accessible. In case a one-on-one interaction is not possible, someone who is accessible by email is good enough. In case you are looking at a surgical grooming, it makes sense to find a center which has a good wetlab and archives of training videos.

A very busy and important teacher may be able to give you tips in an intensive, short mentorship program. This short burst of teaching requires you to be read and receptive at that particular point in time. But, in case you are looking for a holistic, more thorough training program, your best bet would be a teaching hospital with a structured training program. Whereby, several teachers can ensure that your learning is comprehensive and complete.

Very often, a mentorship program headed by a truly brilliant clinician-teacher, supported by an able mid-level supporting crew is best suited for most of our needs. Where the humdrum and the routine is learned by passive and active imbibition of clinical practices and the truly inspirational quality is attained by association with the head mentor.

### Availability of Resources

For many of us, lack of financial support is a major deterrent when choosing a training program. A mentor who can provide part or complete financial aid to support the training is an obvious choice in this case. It is also judicious to apply for local and national societies for help; most offer small to medium term grants to help doctors in training.

### Evaluating the Productivity of the Mentor

Keeping your personal goals in mind, you can evaluate the productivity of the mentorship program you are applying for. In case your aim is to be involved in research and publications, evaluating the projects which the previous mentees have worked on is an easy process. Also, the number of publications from the proposed mentor's team in the last 5 years is an indication of the group's research interests.

If your interest is primarily surgical training, then it is imperative to associate with a high volume practice. Surgeries that percolate down to a surgeon in training are a fraction of the total pool, therefore, it is logical that a busy surgeon will be the best suited for your needs.

### Overseas Training

Get out of your comfort zone. It is a cultural as well as an academic exercise.

## CONCLUSION

A good mentor can help you identify and actualize your career goals. A capable mentor will also enable you target weaknesses, build on leadership skills, and help formulate your career plan. It is therefore, a decision that must be taken judiciously.

To sum it up, look for a ‘Mentor' whose hindsight can become your foresight.
